# Gastroptosis as a Risk Factor of Stent Migration After Endoscopic Ultrasound‐guided Hepaticogastrostomy: A Case Report

**DOI:** 10.1002/deo2.70307

**Published:** 2026-03-02

**Authors:** Wataru Yamagata, Arisa Takeda, Yuki Fuji, Takuya Yokota, Natsuki Miura, Akiko Kitazume, Dai Inoue, Hideyuki Horike, Shigetaka Yoshinaga, Shin Namiki

**Affiliations:** ^1^ Department of Gastroenterology and Hepatology Tokyo Metropolitan Tama Medical Center Tokyo Japan

**Keywords:** EUS‐guided biliary drainage (EUS‐BD), EUS‐guided hepaticogastrostomy (EUS‐HGS), gastroptosis, obstructive jaundice, stent migration

## Abstract

Endoscopic ultrasound‐guided hepaticogastrostomy (EUS‐HGS) has become an established, alternative biliary drainage technique for patients with failed or difficult endoscopic retrograde cholangiopancreatography. Stent migration is a particularly serious adverse event of this method. To minimize the risk of migration, preoperative computed tomography (CT) may be used to confirm the distance between the stomach and the liver. We herein report a rare case of stent migration that occurred despite the use of a long stent. Although preoperative CT demonstrated an acceptable hepaticogastric distance, pronounced gastroptosis involving an excessive, downward extension of the stomach during esophagogastroduodenoscopy on postoperative day 3 led to stent migration. This case highlights the importance of recognizing gastroptosis as a potential risk factor of stent migration following EUS‐HGS.

## Graphical abstract

Gastroptosis may be associated with a higher risk of stent migration than non‐gastroptotic anatomy.

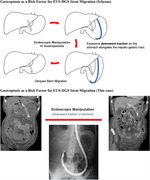

## 1 Introduction

Endoscopic ultrasound‐guided hepaticogastrostomy (EUS‐HGS) is increasingly utilized as an alternative biliary drainage technique for patients in whom endoscopic retrograde cholangiopancreatography (ERCP) proves difficult or unsuccessful. Although the technical success rate has improved over time, procedure‐related adverse events remain a concern. This is particularly true of stent migration, which can lead to serious intraperitoneal complications.

Previous studies have reported various strategies aimed at reducing stent migration, such as preprocedural assessment of the hepatic–gastric distance using computed tomography (CT) [1]; ensuring adequate intragastric stent length [2, 3]; and employing anti‐migration stent designs or deployment techniques [4, 5, 6]. However, these strategies mainly address technical or device‐related factors, and the influence of patient‐specific anatomical variations has not been well characterized.

Gastroptosis, defined as caudal displacement of the stomach, is a relatively common but often underrecognized anatomical condition that can significantly influence the geometry of the hepaticogastric tract, particularly during endoscopic manipulation or dietary intake. However, its possible relevance in EUS‐HGS–related stent migration has not been sufficiently clarified.

We herein present a case in which marked gastroptosis contributed to delayed stent migration after EUS‐HGS, highlighting an anatomical risk factor that may warrant consideration in preprocedural planning and intraprocedural assessment.

## 2 Case Report

**FIGURE 1:**
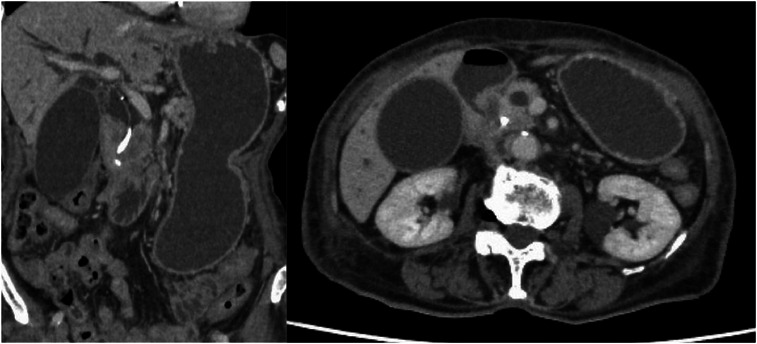
Computed tomography demonstrated an enlarged pancreatic head tumor causing a gastric outlet obstruction (GOO) and malignant biliary obstruction. The stomach and intrahepatic bile ducts were dilated.

A 96‐year‐old female patient presented with obstructive jaundice and nausea. She had pancreatic head cancer and had previously undergone ERCP with placement of a plastic biliary stent for biliary obstruction, after which she opted for best supportive care. CT revealed a gastric outlet obstruction and malignant biliary obstruction due to the pancreatic head tumor (Figure 1).

**FIGURE 2:**
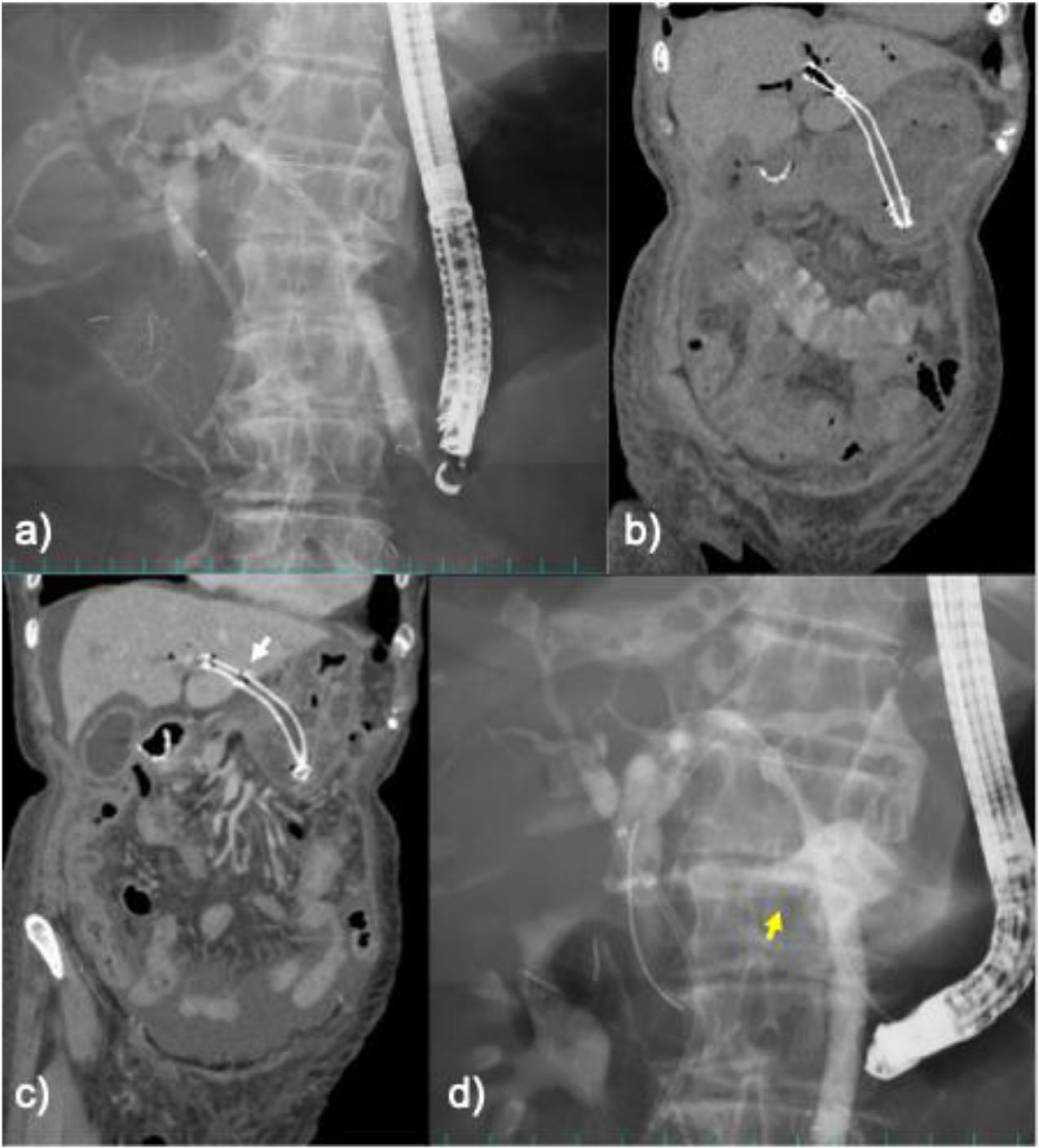
Imaging findings after endoscopic ultrasound‐guided hepaticogastrostomy (EUS‐HGS) and subsequent stent migration. (a) Endoscopic ultrasound‐guided hepaticogastrostomy (EUS‐HGS) with placement of a partially covered self‐expandable metal stent (SEMS) from the B3 duct to the stomach. (b) Postoperative computed tomography confirmed the correct positioning of the partially covered SEMS (PCSEMS) for EUS‐HGS. (c) Three days later, computed tomography demonstrated a slight, distal dislocation of the SEMS on the hepatic side, with the marker between the uncovered and covered portions falling outside the liver margin (white arrow). (d) Cholangiography during the reintervention demonstrated contrast leakage from the uncovered segment of the SEMS into the peritoneal cavity (yellow arrow).

Upper endoscopy with fluoroscopy demonstrated the obstruction extending from the duodenal bulb to the descending duodenum, including the major papilla. An uncovered self‐expandable metal stent (UCSEMS) was deployed from the stomach to the descending duodenum before the EUS‐HGS (Figure S1). Using a curved linear‐array echoendoscope (EG‐740UT; Fujifilm, Tokyo, Japan), the intrahepatic bile duct at segment 3 (B3) was punctured via the lower gastric body with a 19‐gauge FNA needle (EZ Shot 3 Plus; Olympus, Tokyo, Japan) (Figure S2). A 0.025‐inch guidewire (Fielder; Asahi Intecc, Aichi, Japan) was then advanced into the common bile duct, and the segment between the intrahepatic bile duct and the stomach was dilated with a catheter (SHOREN; Kaneka Medix, Osaka, Japan). A partially covered SEMS (PCSEMS, Niti‐S EUS‐BD System 8 mm × 12 cm; Taewoong Medical, Seoul, Korea) was then placed (Figure 2a). On the following day, CT confirmed that the PCSEMS for EUS‐HGS had been placed appropriately (Figure 2b). In contrast, migration of the UCSEMS into the duodenal bulb was suspected on the same CT (Figure S3). Because the EUS‐HGS stent was stable, oral intake was initiated. The patient subsequently experienced nausea, prompting further evaluation.

Upper gastrointestinal endoscopy was performed on postoperative day 3 to evaluate the suspected migration of the UCSEMS rather than as part of routine postoperative follow‐up. Endoscopy confirmed the migration of the UCSEMS into the duodenal bulb. Endoscopy was performed for diagnostic confirmation, and no therapeutic manipulation of the UCSEMS was attempted. After endoscopy, the patient developed abdominal distension, raising concern for a procedure‐related complication. CT was then performed and revealed a slight dislocation of the PCSEMS on the hepatic side, with the marker between the uncovered and covered portions falling outside the liver margin (Figure 2c), a serious adverse event requiring urgent reintervention.

**FIGURE 3:**
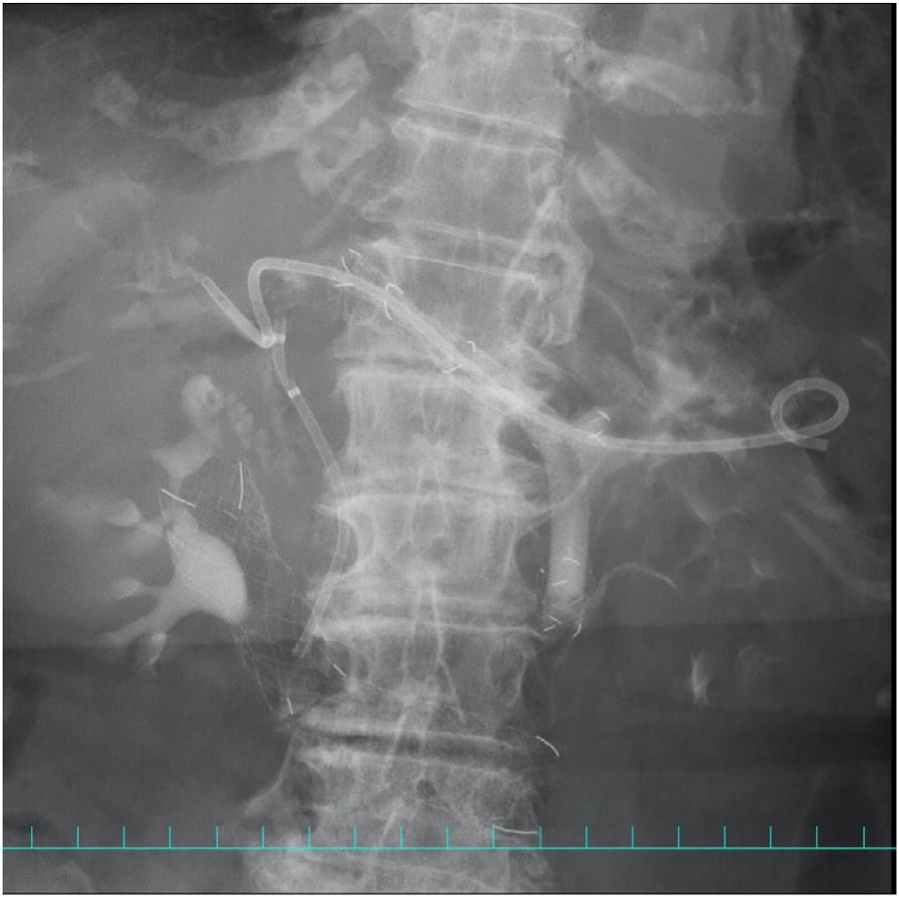
Deployment of a fully covered self‐expandable metal stent (SEMS) across the migrated segment of the partially covered SEMS. A double‐pigtail plastic stent was placed inside the SEMS for anchoring. Cholangiography confirmed resolution of the leak.

A catheter was inserted through the PCSEMS cell near the puncture site and advanced through the existing transgastric fistula, and a guidewire was introduced into the common bile duct. Cholangiography demonstrated contrast leakage into the peritoneal cavity from the uncovered segment of the PCSEMS located just distally to the marker at the junction with the covered portion (Figure 2d). A fully covered SEMS (FCSEMS) was deployed across the migrated segment, and a double‐pigtail plastic stent was placed inside the FCSEMS for anchoring (Figure 3). The leak was resolved immediately, and the patient's free air and cholestatic peritonitis improved promptly after the reintervention.

## 3 Discussion

EUS‐guided biliary drainage (EUS‐BD) has recently gained acceptance as an alternative drainage method in patients who experience difficulty with ERCP, including those with a malignant distal biliary obstruction or surgically altered anatomy. Moond et al. were the first to conduct a meta‐analysis of EUS‐HGS, which found technical and clinical success rates of 94.4% and 88.6%, respectively. The rates of mild, moderate, severe, and fatal adverse events were 5.8%, 12.1%, 4.2%, and 3.7%, respectively, and stent migration occurred in 5.3% of the cases [7]. Although these rates may vary by region, stent migration remains one of the most serious complications of EUS‐HGS [8, 9].

Several preventive strategies have been proposed. Ochiai et al. emphasized confirming the gastric–hepatic distance on preoperative CT as a means of selecting an appropriate stent length [1]. Nakai et al. recommended positioning more than 5 cm of the stent within the stomach [2]. Miyano et al. proposed an intra‐scope channel release technique to reduce the migration risk [3] while other studies have anchored the stents using clips or deployed plastic stents [4, 5]. Newly designed stents with anchoring features have also recently been developed [6].

**FIGURE 4:**
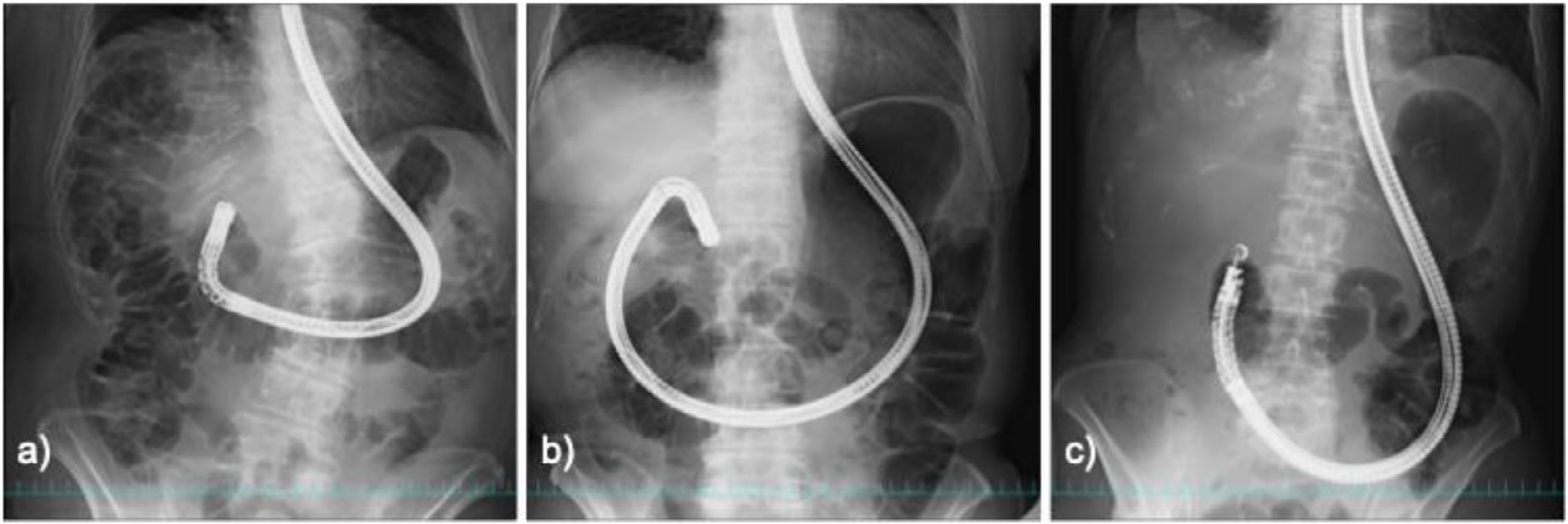
Fluoroscopic patterns of gastric distension during full‐push insertion of the scope into the pylorus. (a) Stable pattern with minimal distension. (b) Upper pattern with distension extending to the upper pelvic margin. (c) Inner pattern with distension extending into the pelvic cavity consistent with gastroptosis.

Our patient had marked gastroptosis. Scope manipulation had displaced the stomach downward into the pelvis, extending the hepatic–gastric distance beyond that estimated on CT and thereby dislocating the PCSEMS. Unlike the commonly reported mechanisms of migration during stent release or direct scope–stent interference, this case demonstrated a rare pattern of delayed migration triggered by endoscopic manipulation in the context of gastroptosis. A review of the fluoroscopic images obtained during endoscopy revealed the “inner pattern” of gastric distension, in which the stomach extends into the pelvic cavity (Figure 4c). This finding, which is consistent with gastroptosis, was not fully appreciated at the time of the procedure. In addition to gastroptosis, factors such as the puncture site and dynamic gastric movement during endoscopic manipulation likely contributed to the elongation of the hepatogastric tract and subsequent stent migration.

Although preprocedural CT demonstrated an acceptable hepatic–gastric distance, the ability of this modality to assess gastroptosis is limited because imaging is performed in the supine position and in a fasted state and does not reflect dynamic gastric positioning during endoscopic manipulation. Therefore, to minimize the risk of stent migration, it is important not only to evaluate the hepatic–gastric distance on preoperative CT but also to assess the gastric configuration via fluoroscopy and endoscopy.

Three fluoroscopic patterns of gastric distension with full‐push insertion into the pylorus were identified during ERCP/EUS: (1) a stable pattern without significant distension; (2) an upper pattern with distension to the upper pelvic margin; and (3) an inner pattern with distension into the pelvic cavity (Figure 4). Patients with the inner pattern may have a higher risk of stent dislocation due to gastroptosis.

It is well recognized that the fistula is unstable in the early postoperative period and that early upper gastrointestinal endoscopy itself is a known risk factor for EUS‐HGS stent migration; therefore, endoscopy should be avoided whenever possible within the first few days after EUS‐HGS to prevent stent displacement. In retrospect, given the necessity of early endoscopy in this case, a repeat CT scan and careful assessment of the HGS stent position should have been performed beforehand. We emphasize that strict pre‐endoscopic assessment is crucial, especially in patients with potential gastroptosis, as gastric distension during the procedure can significantly elongate the hepaticogastric distance. The design of PCSEMS, particularly on the proximal side, also needs to be improved to reduce the migration risk. Finally, the puncture site should be carefully chosen using EUS‐HGS. In patients with gastroptosis, puncturing the gastric body at a high rather than distal site may help reduce the hepatic–gastric distance and minimize the risk of stent migration.

Taken together, our findings suggested that gastroptosis may contribute to stent migration after EUS‐HGS. Careful assessment of the gastric configuration and appropriate selection of the puncture site may help reduce this risk.

## Author Contributions

**Wataru Yamagata** gathered the patient data and wrote the manuscript. **Wataru Yamagata**, **Arisa Takeda**, and **Yuki Fuji** were responsible for managing the patient. **Wataru Yamagata**, **Takuya Yokota**, **Natsuki Miura**, and **Hideyuki Horike** performed the EUS‐HGS and reintervention. **Akiko Kitazume** and **Dai Inoue** participated in the literature review and manuscript revision. **Shigetaka Yoshinaga** contributed to the critical revision of the manuscript. **Shin Namiki** supervised clinical operations as the department head. All the authors have contributed substantially to the revision of the manuscript, whose content is solely the responsibility of the authors.

## Funding

The authors have nothing to report.

## Conflicts of Interest

The authors declare no conflicts of interest.

## Supporting information




**Figure S1** UCSEMS placement across the pylorus for gastric outlet obstruction (GOO). (a) Upper endoscopy with fluoroscopy demonstrated GOO extending from the duodenal bulb to the second portion of the duodenum. (b) An uncovered self‐expandable metal stent (UCSEMS) was deployed from the stomach to the second portion of the duodenum for the gastric outlet obstruction. (c) Endoscopic view showing the UCSEMS in situ, with its proximal end positioned beyond the pylorus. (d) On postoperative day 3, the UCSEMS was found to have migrated into the duodenal bulb.

**Figure S2** EUS‐guided hepaticogastrostomy (EUS‐HGS). (a) Real‐time EUS image showing puncture of the left intrahepatic bile duct (B3) via the lower gastric body using a 19‐gauge FNA needle. (b) Fluoroscopic cholangiography obtained after puncturing the left intrahepatic bile duct (B3). (c) Endoscopic view showing the PCSEMS for EUS‐HGS via the lower gastric body.

**Figure S3** CT images obtained on the day after EUS‐HGS. Panels (a)—(d) show sequential slices from anterior to posterior. These findings are suggestive of possible UCSEMS migration into the duodenal bulb.
