# Supplementary figures and images for: Gastroptosis as a Risk Factor of Stent Migration After Endoscopic Ultrasound‐guided Hepaticogastrostomy: A Case Report
Source: DEN Open. 2026 Mar 2;6(1):e70307. doi: 10.1002/deo2.70307 (PMC12953050; doi:10.1002/deo2.70307)

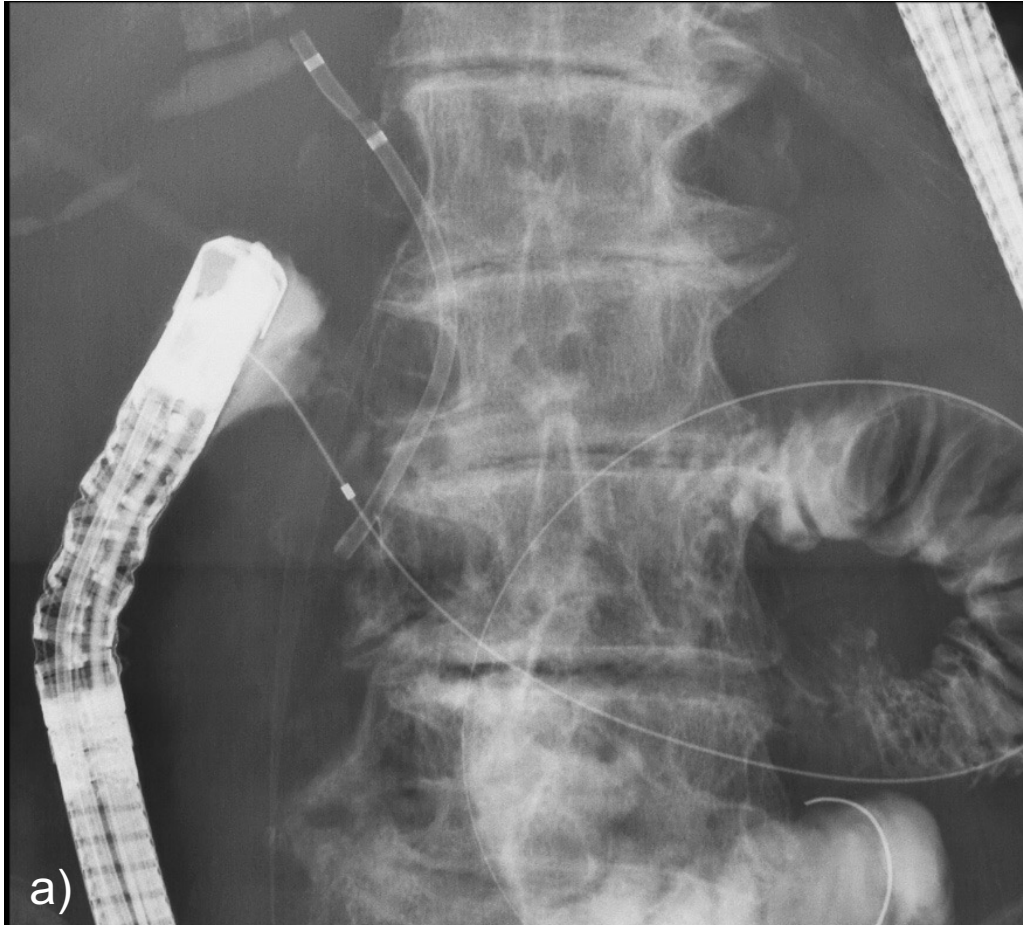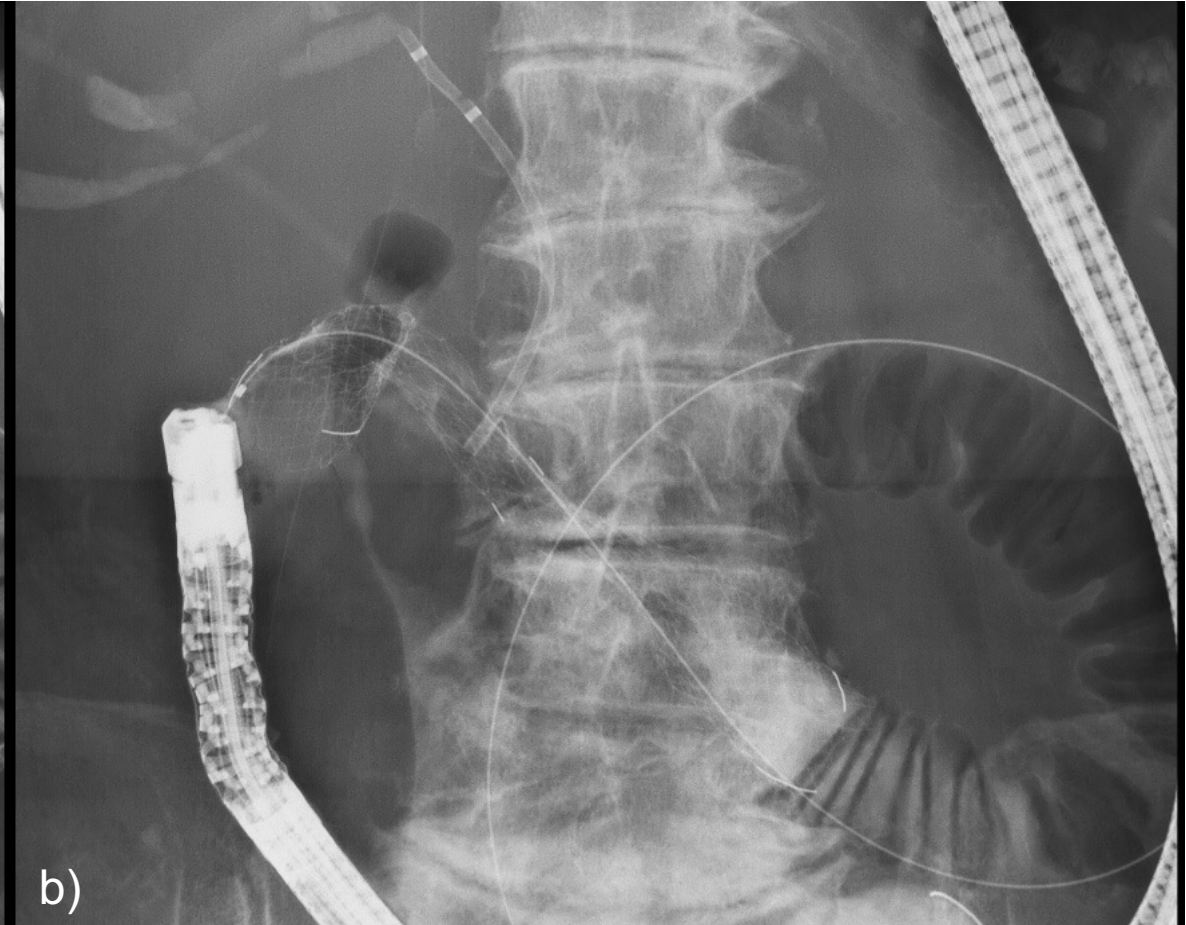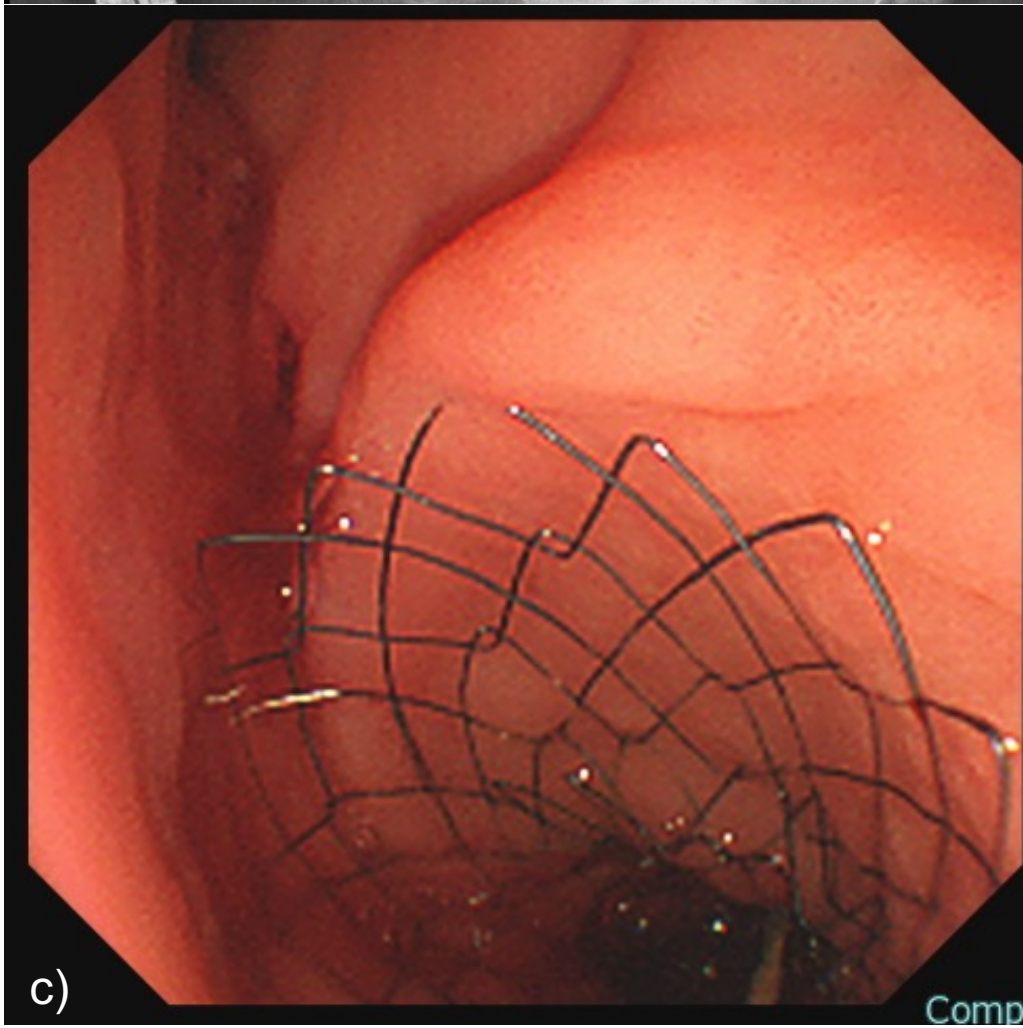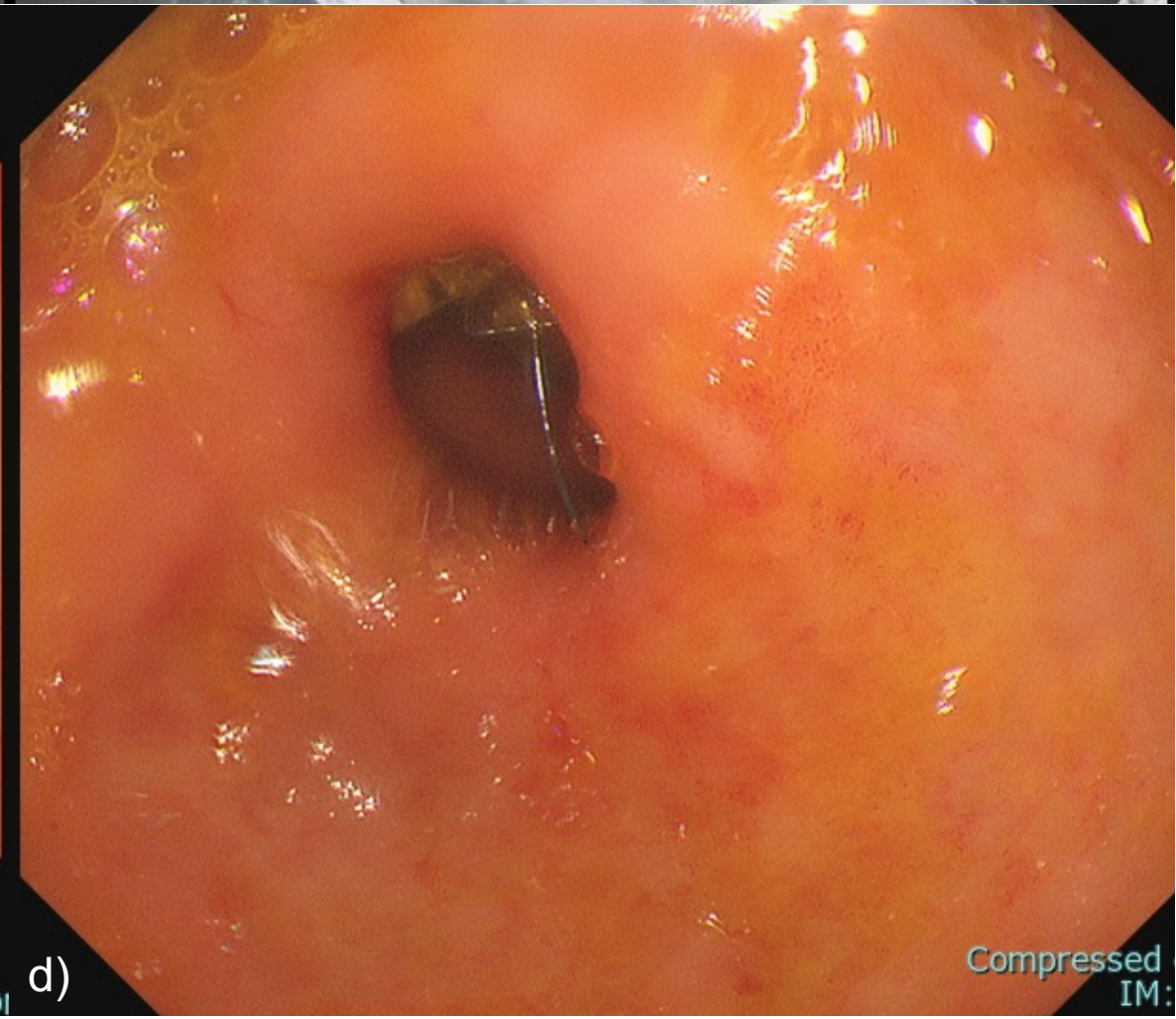

Compi

Compressed  
IM:

Supplement: Supplementary file 1 — Figure S1 UCSEMS placement across the pylorus for gastric outlet obstruction (GOO). (a) Upper endoscopy with fluoroscopy demonstrated GOO extending from the duodenal bulb to the second portion of the duodenum. (b) An uncovered self‐expandable metal stent (UCSEMS) was deployed from the stomach to the second portion of the duodenum for the gastric outlet obstruction. (c) Endoscopic view showing the UCSEMS in situ, with its proximal end positioned beyond the pylorus. (d) On postoperative day 3, the UCSEMS was found to have migrated into the duodenal bulb. [file DEO2-6-e70307-s003.pdf]

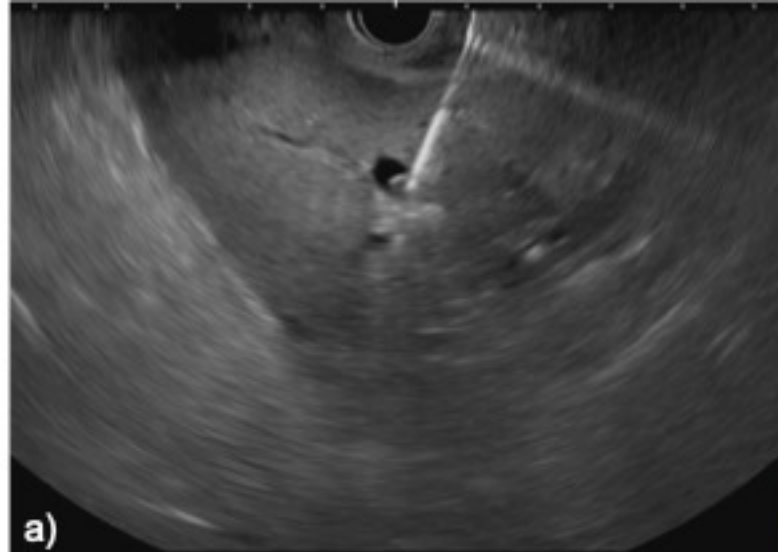

a)

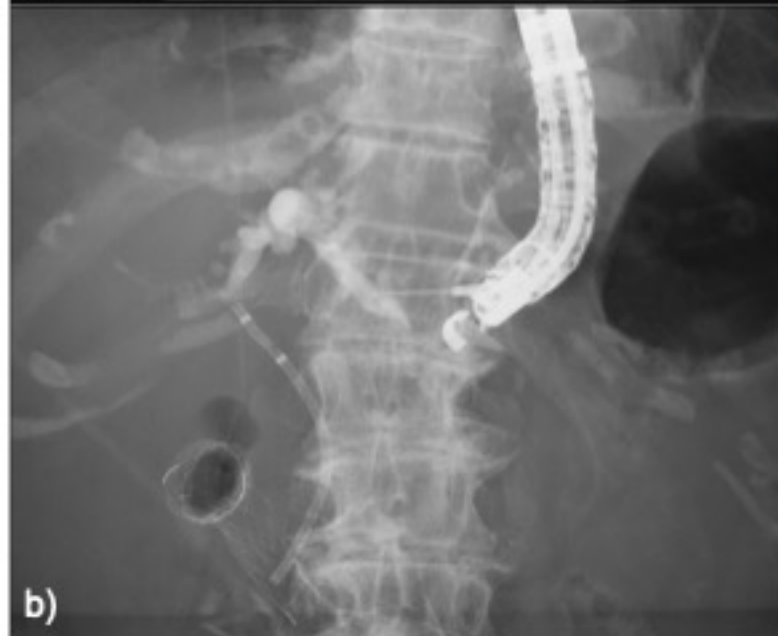

b)

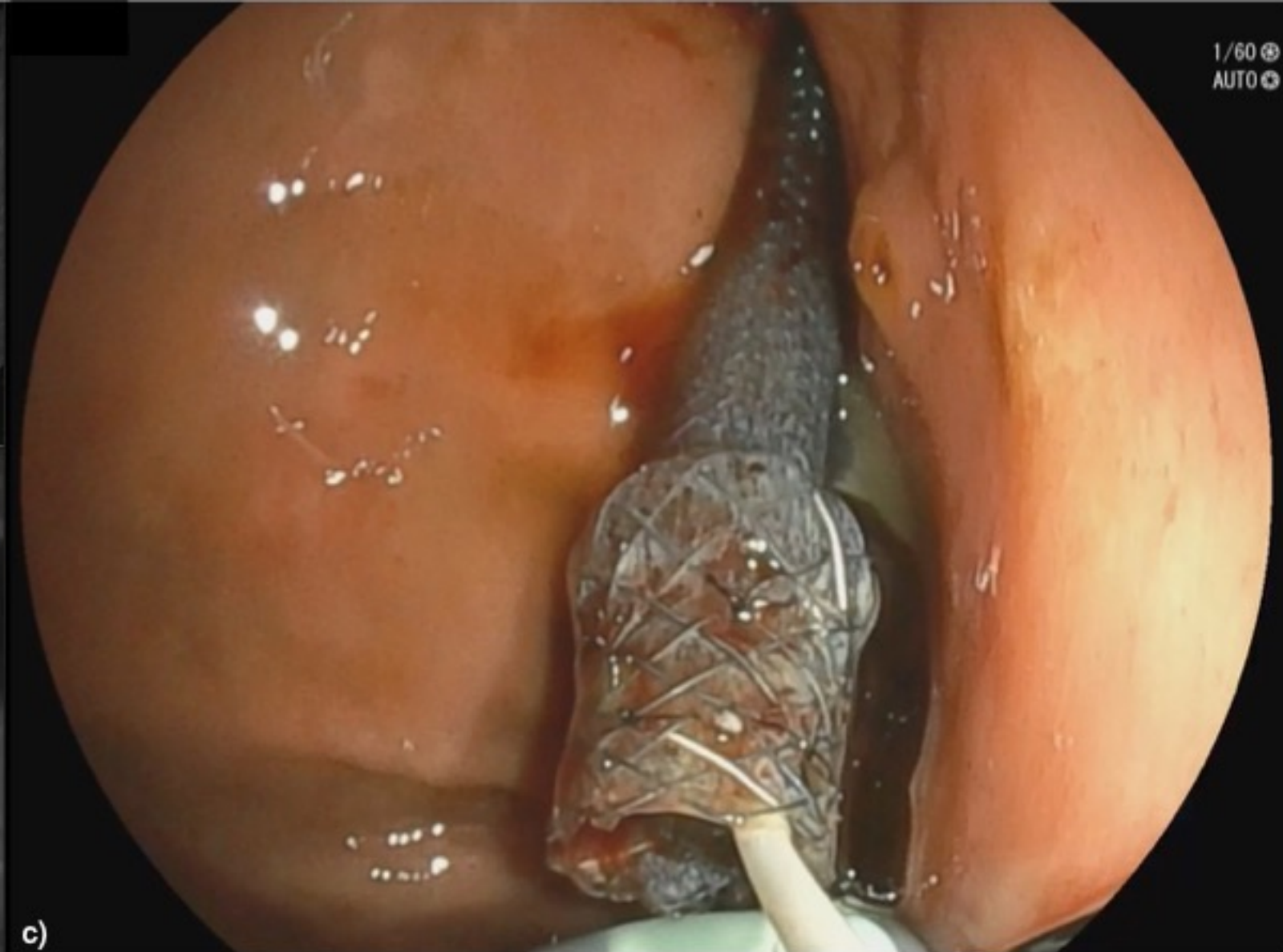

c)

Supplement: Supplementary file 2 — Figure S2 EUS‐guided hepaticogastrostomy (EUS‐HGS). (a) Real‐time EUS image showing puncture of the left intrahepatic bile duct (B3) via the lower gastric body using a 19‐gauge FNA needle. (b) Fluoroscopic cholangiography obtained after puncturing the left intrahepatic bile duct (B3). (c) Endoscopic view showing the PCSEMS for EUS‐HGS via the lower gastric body. [file DEO2-6-e70307-s002.pdf]

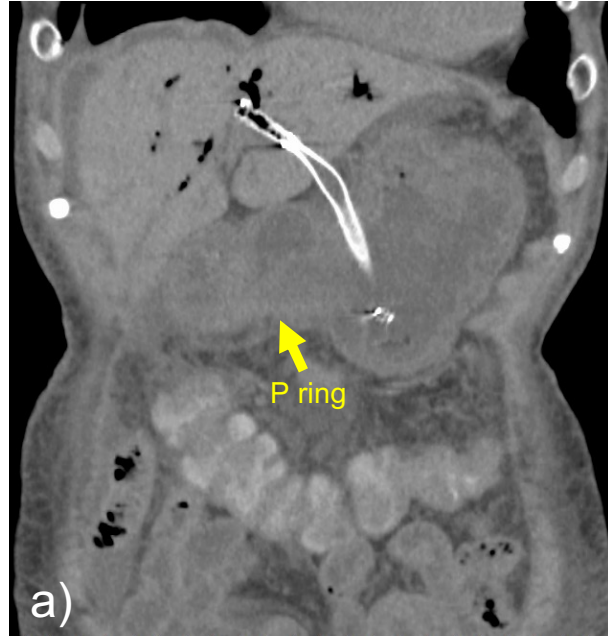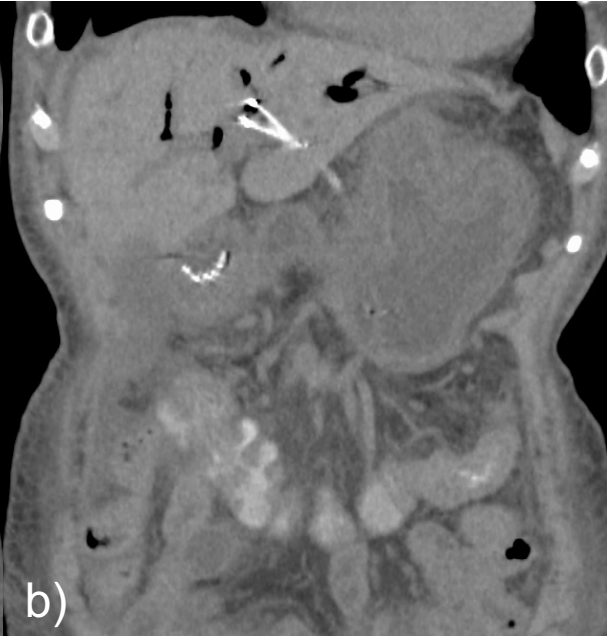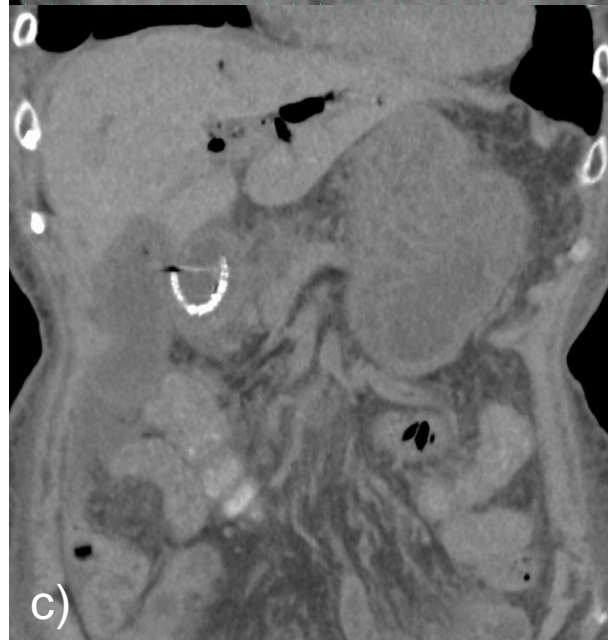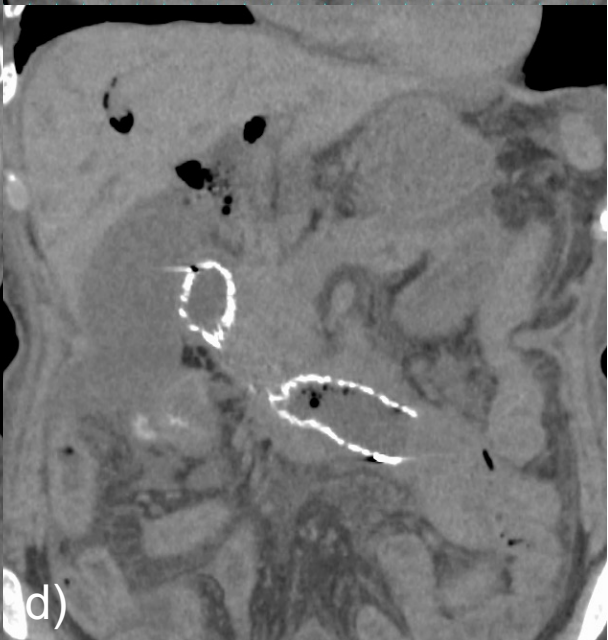

Supplement: Supplementary file 3 — Figure S3 CT images obtained on the day after EUS‐HGS. Panels (a)—(d) show sequential slices from anterior to posterior. These findings are suggestive of possible UCSEMS migration into the duodenal bulb. [file DEO2-6-e70307-s001.pdf]
